# Aggressive Fibromatosis: Evidence for a Stable Phase

**DOI:** 10.1080/13577149877902

**Published:** 1998-12

**Authors:** Gillian Mitchell, J. Meirion Thomas, Clive L. Harmer

**Affiliations:** Sarcoma Unit Royal Marsden Hospital NHS Trust Fulham Road London SW3 6JJ UK

## Abstract

*Purpose.* Aggressive fibromatosis (AF) is an uncommon locally infiltrating benign disease of soft tissue for which treatment comprises complete surgical resection. Radiotherapy can be given postoperatively if the margin is incompletely resected. If the tumour is inoperable radiotherapy provides an alternative treatment. Hormone therapy and cytotoxic chemotherapy have also been used for unresectable or recurrent disease. All treatment modalities carry an associated morbidity. We believe that the natural history of aggressive fibromatosis may include a period of stable disease without progression, during which time, treatment is not always necessary.

*Patients and methods.* We present a retrospective review of 42 patients referred to the Royal Marsden Hospital between 1988 and 1995 with aggressive fibromatosis. Evidence of periods of stable disease and the relationship to delivered treatment was obtained from the case notes, including the natural history prior to referral to our institution. Stable disease was defined as a period of no objective progression for 6 months or longer.

*Results.* Seventeen patients could be assessed for stable disease and all (100%) experienced at least one episode of stable disease, eight of whom whilst receiving hormonal or cytotoxic therapy. Of the 23 patients who could not be assessed for stable disease, as they underwent surgery at presentation or recurrence of disease, only 2 had persisting disease at last follow-up. Both of these patients had had positive surgical resection margins.

*Discussion.* This study demonstrates the variable natural history of AF, which can include a substantial period of stable disease in a significant number of patients. A less aggressive approach to the management of AF may therefore be appropriate, particularly if a subgroup of patients who are likely to experience a period of stable disease can be identified.


*Sarcoma* (1998) **2**, 149–154

ORIGINAL ARTICLE

### Aggressive fibromatosis: evidence for a stable phase

GILLIAN MITCHELL, J. MEIRION THOMAS & CLIVE L. HARMER

*Sarcoma Unit, Royal Marsden Hospital NHS Trust, London SW3 6JJ, UK*

**Abstract**

*Purpose.* Aggressive fibromatosis (AF) is an uncommon locally infiltrating benign disease of soft tissue for which treatment comprises complete surgical resection. Radiotherapy can be given postoperatively if the margin is incompletely resected. If the tumour is inoperable radiotherapy provides an alternative treatment. Hormone therapy and cytotoxic chemotherapy have also been used for unresectable or recurrent disease. All treatment modalities carry an associated morbidity. We believe that the natural history of aggressive fibromatosis may include a period of stable disease without progression, during which time, treatment is not always necessary.

*Patients and methods.* We present a retrospective review of 42 patients referred to the Royal Marsden Hospital between 1988 and 1995 with aggressive fibromatosis. Evidence of periods of stable disease and the relationship to delivered treatment was obtained from the case notes, including the natural history prior to referral to our institution. Stable disease was defined as a period of no objective progression for 6 months or longer.

*Results.* Seventeen patients could be assessed for stable disease and all (100%) experienced at least one episode of stable disease, eight of whom whilst receiving hormonal or cytotoxic therapy. Of the 23 patients who could not be assessed for stable disease, as they underwent surgery at presentation or recurrence of disease, only 2 had persisting disease at last follow-up. Both of these patients had had positive surgical resection margins.

*Discussion.* This study demonstrates the variable natural history of AF, which can include a substantial period of stable disease in a significant number of patients. A less aggressive approach to the management of AF may therefore be appropriate, particularly if a subgroup of patients who are likely to experience a period of stable disease can be identified.

**Key words**: *aggressive fibromatosis, stable growth phase.*

### Introduction

Aggressive fibromatosis is an uncommon benign soft tissue neoplasm. Because of its tendency for recurrence, it is usually categorised together with malignant soft tissue neoplasms of which it constitutes less than 4%.[1] The reported annual incidence is 0.2–0.5/100 000 population.[2] It occurs more commonly in women and in the younger population. Aetiology is unknown but there are associations with trauma (including surgery), pregnancy, and oestrogen exposure. There is a rare familial type, which may be part of Gardner's syndrome.

Fibromatosis was first described more than 150 years ago by McFarlane.[2] Because of its rarity, most published series describe only small numbers of patients and there remains uncertainty as to the most appropriate method of treatment. Current approaches include surgery, radiotherapy, chemotherapy, endocrine therapy and observation, either alone or in combination.

Assessment of response to therapy has traditionally included only local control and recurrence rates. However, there is the impression in our institution that there may be a plateau phase in its natural history when there is no progression, i.e. a period of stable disease. If this exists then there are serious implications for management, particularly when treatments can involve either extensive surgery or potentially mutagenic treatments such as radiotherapy, chemotherapy and endocrine therapy. Furthermore, if there is a plateau phase then it must be taken into account when assessing response to treatment. Growth arrest with any of these treatments may be unrelated to that treatment and failure to progress (stable state) cannot be considered an objective response.

### Materials and methods

A retrospective analysis was made of 42 evaluable patients referred to one surgeon at the Royal Marsden Hospital (RMH), with a diagnosis of aggressive fibromatosis between 1988 and 1995. Twenty-one patients were referred with recurrent disease after treatment elsewhere and 21 presented *de novo*. A

Correspondence to: G. Mitchell, Sarcoma Unit, Royal Marsden NHS Trust, Fulham Road, London SW3 6JJ, UK. Fax: 0181 770 1489.

1357-714X/98/030149-06 $9.00 © 1998 Carfax Publishing Ltd

**Table 1**. *Tumour site in patients with or without stable disease (SD)*

| | Site | | | |
|---|---|---|---|---|
| | Abdominal wall | Intra-abdominal | Pelvis | Others |
| Patients with SD | 1 | 1 | 3 | 14 |
| Patients without SD | 3 | 3 | 1 | 16 |

range of treatments was offered from observation only, surgery alone, surgery and radiotherapy (30–60 Gy in 15–30 fractions given over 3–6 weeks), toremifene 40 mg daily, or chemotherapy with methotrexate (50 mg weekly) plus vinblastine (10 mg weekly). A wide excision was the aim of surgery, if practically possible. Wide excision is the removal of all gross disease with a surrounding margin of healthy tissue. Follow up ranged from 11 months to 8 years (median 3 years). Twelve patients were not followed up, but the natural history of their disease prior to referral is included in the analysis. Follow-up data are available on 30 patients.

Stable disease was defined as a period of no objective progression for 6 months or longer. Evidence of stable disease was obtained from case notes, either reported by patients in their initial history or by documented reports during follow up at RMH. Progressive disease was defined as an increase in size or symptoms related to the tumour. Complete response was resolution of all gross disease following therapy. Partial response was a reduction in size of the tumour by 50% or greater in all dimensions maintained for two consecutive assessments at least 3 months apart. Residual disease was defined as microscopic, if all gross disease was removed but margins were positive on microscopy, or macroscopic, if gross disease remained. The periods of progressive, stable or regressing disease were then correlated with the type of treatment administered.

### Results

The case notes of 42 patients with aggressive fibromatosis seen at RMH were retrospectively analysed. Twenty nine patients were female and 13 male. The age range at the time of referral was 16–75 years (median 34 years). Of the patients who presented with recurrent disease, 2 were found to have scar tissue only, 8 patients had developed their first recurrence and 11 had undergone multiple excisions with or without radiotherapy. Six patients had 2 or more sites of disease. The anatomical sites of involvement are shown in Table 1.

### *Episodes of stable disease*

Episodes of stable disease were seen in 17 patients (40%). Fifteen patients had stable disease documented following referral to RMH, 4 of these also had stable disease prior to RMH referral. Only two patients had stable disease that was not observed at RMH but had been documented by the referring hospital. Spontaneous regression was seen in 2 additional patients. One patient with stable disease also experienced spontaneous regression at one site and experienced stable disease at another, separate, site. Table 2 summarises these data. Twelve patients were female and 7 male. The period of stable disease ranged from 6 months to 12 years (median 3 years). Nine of these patients had periods of stable disease on no treatment. Eight patients had stable disease while on treatment. Seven were being treated with toremifene (20 mg daily). Three patients were also treated with methotrexate plus vinblastine and one patient with chemotherapy alone.

Four of these patients continued to have stable disease after completion of therapy from 21 to 36 months and 4 have continued to have stable disease while continuing with therapy. Three patients experienced a spontaneous regression.

### *No episode of stable disease*

Twenty-three patients could not be assessed for stable disease as they were treated by surgery. All but two or these had recurrent disease at referral. Twelve patients underwent surgery alone, 5 received surgery and radiotherapy, 3 commenced toremifene and one was started on chemotherapy. The latter 4 patients progressed on drug therapy and one of these had a further resection and remains disease free. Table 3 outlines outcome after surgery in relation to the histological excision margin in patients treated with surgery with or without radiotherapy. Five patients had surgery followed by adjuvant radiotherapy for positive histological margins. Follow-up data are available on only two of these: both remain free of disease 3 and 4 years post-irradiation. The final three patients were not treated at RMH having been discharged after their initial consultation.

In summary, of these 23 patients, only 2 still had persisting disease at their last follow-up, despite the presence of gross residual disease or positive resection margins in 10 (13 if unknown excision margins are included). Both patients with persisting disease had previously relapsed on more than one occasion

**Table 2**. *Characteristics of patients with stable disease, duration of stable disease and relationship to treatment. S = surgery, RT = radiotherapy, Mo = months, Obs = observation, T = toremifene, C = chemotherapy, spont res = spontaneous resolution*

| Patient No. | Age at referral | Gender | Site | Duration of disease | Previous failure | Previous treatment | Duration and setting of stable disease | |
|---|---|---|---|---|---|---|---|---|
| | | | | | | | During observation | During treatment |
| 1 | 52 | M | leg | 23y | multiple | S,RT | 8y | x |
| 2 | 59 | F | trapezius | 3y | no | none | 3y | x |
| 4 | 43 | M | scapula | 10y | no | none | 10y | x |
| 7 | 26 | F | leg | 3y | no | none | 3y | x |
| 22 | 17 | F | abdominal wall | 10Mo | no | none | 10Mo | x |
| 24 | 66 | F | scapula | not recorded | no | none | x | 2y on T |
| 25 | 18 | F | retroperitoneum | 1Mo | no | none | 2y | x |
| 26 | 31 | F | pelvis | 6y | yes | S | x | 3y on T |
| 27 | 26 | F | leg | 18Mo | no | none | x | 3y on T |
| 29 | 29 | M | scapula | 15y | yes | RT | 15y | x |
| 30 | 51 | M | trapezius | not recorded | no | none | x | 42Mo on C, T, Obs |
| 31 | 20 | M | arm | 2y | yes | S | x | 39 Mo on T, Obs |
| 32 | 34 | F | leg | 8y | multiple | S,RT | x | 46Mo on C, Obs |
| 34 | 30 | F | pelvis | 1Mo | no | none | x | 3y on T, C, Obs |
| 35 | 18 | F | neck | 3Mo | no | none | 20Mo | x |
| 36 | 43 | M | leg | 2Mo | no | none | 8Mo | x |
| 37 | 34 | F | pelvis | 1Mo | no | none | 2y then spont res | x |
| 41 | 75 | M | leg | 3Mo | no | none | 15Mo | x |
| | | | leg | 3Mo | no | none | 15Mo then spont res | x |
| 42 | 28 | F | leg | 2Mo | no | none | 3Mo then spont res | x |

**Table 3.** *Disease outcome related to status of surgical margins in patients treated by surgery with or without radiotherapy: x = no follow-up*

| Patient No. | Histological margins | Disease status | FU (months) |
|---|---|---|---|
| 3 | micro positive | x | x |
| 5 | gross disease | recurrence | 5 |
| 6 | gross disease | disease free | 30 |
| 8 | clear | x | x |
| 9 | clear | disease free | 1 |
| 10 | not reported | disease free | 72 |
| 11 | not reported | disease free | 35 |
| 12 | clear | disease free | 66 |
| 13 | clear | disease free | 11 |
| 14 | gross disease | disease free | 11 |
| 15 | clear | disease free | 23 |
| 16 | micro positive | disease free | 25 |
| 17 | micro positive | disease free | 38 |
| 18 | micro positive | disease free | 50 |
| 19 | micro positive | x | x |
| 20 | micro positive | x | x |
| 21 | micro positive | x | x |
| 23 | not reported | disease free | 26 |

and had gross residual disease following their most recent resection.

### Discussion

The fibromatoses comprise a number of distinct clinicopathological types: palmar and plantar fibromatosis, penile fibromatosis (Peyronie's disease) and aggressive fibromatosis (desmoid tumour). The latter are divided into abdominal or extra-abdominal sites. The abdominal tumours usually occur in the muscular aponeurotic structures of the anterior abdominal wall, particularly in post-partum women. Extra-abdominal tumours occur at any site but are more usual around the shoulder girdle, inguinal region and lower extremities.

Histologically the tumour consists of interlacing bundles of spindle cells (fibroblasts) with variable amounts of collagen, which infiltrate the surrounding structures. There can be vigorous cellularity at the edge of the lesion in comparison to a relatively poorly cellular core and this, together with the tendency to infiltrate and a high tendency to recurrence post-excision, has led some authors to describe the condition as low-grade fibrosarcoma.[3,4] Metastases do not occur.

The importance of aggressive fibromatosis is a tendency to infiltrate widely which has led most practitioners to treat this tumour in a manner similar to a low-grade sarcoma, by performing a wide excision. The risk of local recurrence after non-wide resection is 8–70%.[1,5,6,7] Multiple previous recurrences and residual disease predict for further recurrence. However, there are also reports of lack of recurrence or progressive disease in cases that are known to have had either gross residual disease or microscopically positive margins at surgery.[3,8,9,10] This implies that the persistence of tumour cells does not necessarily equate with progressive disease. This has been confirmed in our series.

Other modalities used in the management of this disease include radiotherapy, hormone therapy and chemotherapy. Assessment of response includes recurrence rates or response rates (including partial and complete response) and stable disease. In our series 40% of patients had stable disease either with or without treatment. Therefore it is important to identify in reported series the type of response in order to assess the possible benefit of treatment.

Radiotherapy is frequently recommended in both the primary and adjuvant setting. Radiotherapy series have shown a local recurrence rate of 20–46%.[4,5,7,9–16] Some series indicate the proportion of complete, partial and stable disease response to treatment, which is summarised in Table 4, although the majority describes complete response or persisting disease only.

The reported use of toremifene and chemotherapy, in the management of this disease is limited.[17–21] Reports describe small numbers of patients and response rates are quoted as 65–75%, but include stable disease, partial and complete response. In our series, 8 patients (67%) had stable disease while using toremifene or chemotherapy, but no patient had a partial or complete response.

It can be seen that there is a wide variation in the natural history of aggressive fibromatosis, ranging from spontaneous remission to multiple recurrences post-treatment, regardless of the modality of treatment used. From our results, 40% of patients have experienced a period of stable disease. The variability of natural history has significant implications for management in the individual patient. The majority of patients will be successfully treated with

**Table 4**. *Aggressive fibromatosis treated by radiotherapy: response to treatment. x = no data, n = number of patients in each category*

| Reference | Patients (*n*) | Complete response (*n*) | Partial response (*n*) | Stable disease (*n*) | Progressive disease (*n*) |
|---|---|---|---|---|---|
| 1 | 10 | 5 | 3 | x | 2, recurrence |
| 7 | 4 | 4 | x | x | x |
| 9 | 24 | 15 | 3, controlled | x | x |
| 10 | 8 | 6 | 2, disease persistence | x | x |
| 11 | 13 | 9 | 4, residual disease | x | x |
| 12 | 16 | x | 14, local control | x | 2 |
| 13 | 14 | 10 | x | x | 4 |
| 14 | 21 | 15 | 2 | 4 | x |
| 15 | 13 | 7 | 1, local persistence | x | 5, recurrence |
| 16 | 8 | 2 | 2 | 4 | x |

wide resection. Adjuvant radiotherapy can be reserved for the minority with a significant risk of recurrence, particularly those with residual bulk disease or multiple previous recurrences. A proportion of patients will be surgically incurable and options for treatment include debulking surgery, radiotherapy, hormone therapy or chemotherapy, if symptoms demand. Most options have potentially serious adverse effects, most importantly induction of a second malignancy. It is our opinion that a significant proportion of these patients may reach a stable phase of their disease and hence not require further treatment with agents that may have serious long-term toxicity. The difficulty is to identify these patients. This study has not revealed any particular features (other than multiple recurrences) which are less likely to have a stable phase.

### Conclusion

The natural history of aggressive fibromatosis is variable and can include a substantial period of stable disease in a significant proportion of patients. Aggressive fibromatosis rarely causes life-threatening complications, although it may be fatal.[22] Hence, we have adopted a non-aggressive approach to management following attempted wide excision. We initially observe unresectable or recurrent disease on this 'stable phase' principle. Debulking surgery is frequently of value for palliation. We now have a series of patients where the tumour has been debulked to achieve a good cosmetic and functional result. We tend to avoid the use of radiotherapy even when resection is incomplete or if the margins are positive, unless recurrence or further progression could have a potentially serious outcome.

### References

1 Keil KD, Suit HD. Radiation therapy in the treatment of aggressive fibromatoses. *Cancer* 1984; 54:(10), 2051–5.

2 Macfarlane J. *Clinical reports on the surgical practice of the Glasgow Royal Infirmary*. Glasgow: D. Robertson, 1832:63–6.

3 Posner MC, Shiu MH, Newsome JL, Hajdu SI, Gaynor JJ, Brennan MF. The desmoid tumour. Not a benign disease. *Arch Surg* 1989; 124:191–6.

4 McKinnon JG, Neifeld JP, Kay S, Parker GA, Foster WC, Lawrence WL. Management of desmoid tumours. *Surg Gynaec Obstet* 1989; 169:104–6.

5 Plukker JT, van Oort I, Vermey A, Hoekstra HJ, Panders AK, Dolsma WV, *et al.* Aggressive bromatosis (non-familial desmoid tumour): therapeutic problems and the role of adjuvant radiotherapy. *Br J Surgery* 1995; 82:510–14.

6 Easter DW, Halasz NA. Recent trends in the management of desmoid tumours. *Ann Surg* 1989; 210:(6), 765–9.

7 Keus R, Bartelink H. The role of radiotherapy in the treatment of desmoid tumours. *Radiother Oncol* 1986; 1–5.

8 Reitamo JJ, Scheinin TM, Hayry P. The desmoid syndrome. New aspects in the cause, pathogenesis and treatment of the desmoid tumour. *Am J Surgery* 1986; 151:230–37.

9 Miralbell R, Suit H, Mankin HJ, Zuckerberg LR, Stracher MA, Rosenberg AE. Fibromatoses: from postsurgical surveillance to combined surgery and radiation therapy. *Int J Radiat Oncol Biol Phys* 1990; 18:535–40.

10 Catton CN, O'Sullivan B, Cummings B, Fornasier V, Panzarella T. Aggressive fibromatosis: optimisation of local management with a retrospective failure analysis. *Radiat Oncol* 1995; 34:17–22.

11 Stockdale AD, Cassoni AM, Coe MA, Phillips RH, Newton KA, Westbury G, *et al.* Radiotherapy and conservative surgery in the management of musculo-aponeurotic fibromatosis. *Int J Radiat Oncol Biol Phys* 1988; 15:851–7.

12 McCollough WM, Parsons JT, Van Der Griend R, Enneking WF, Heare T. Radiation therapy for aggressive fibromatosis. *J Bone Joint Surg* 1991; 73A:(5), 717–25.

13 Sherman NE, Romsdahl M, Evans H, Zagars G, Oswald MJ. Desmoid tumours: a 20-year radiotherapy experience. *Int J Radiat Oncol Biol Phys* 1990; 19:37–40.

14 Schmitt G, Mills EED, Levin V, Smit BJ, Boecker H, Pape H. Radiotherapy of aggressive fibromatosis. *Eur J Cancer* 1992; 28A:(4), 832–5.

15 Leibel SA, Wara WM, Hill DR, Bovill EG, De Lorimier AA, Beckstead JH, *et al.* Desmoid tumours: local control and patterns of relapse following radiation therapy. *Int J Radiat Oncol Biol Phys* 1983; 9:1167–71.
16 Greenberg HM, Goebel R, Weichselbaum RR, Greenberger JS, Chaffey JT, Cassady JR. Radiation therapy in the treatment of aggressive fibromatoses. *Int J Radiat Oncol Biol Phys* 1981; 7:305–10.
17 Kinzbrunner B, Ritter S, Domingo J, Rosenthal J. Remission of rapidly growing desmoid tumours after tamoxifen therapy. *Cancer* 1983; 52:2201–4.
18 Wilcken N, Tattersall MHN. Endocrine therapy for desmoid tumours. *Cancer* 1991; 68:1384–8.
19 Brooks MD, Ebbs SR, Colletta AA, Baum M. Desmoid tumours treated with triphenylethylenes. *Eur J Cancer* 1992; 28A:1014–18.
20 Patel SR, Evans HL, Benjamin RS. Combination chemotherapy in adult desmoid tumours. *Cancer* 1993; 72:3244–7.
21 Weiss AJ, Lackman RD. Low-dose chemotherapy of desmoid tumours. *Cancer* 1989; 64:1192–4.
22 Ashby MA, Harmer CL, McKinna JA, Lennox SC. Case report: Infiltrative fibromatosis: a rare cause of fatal haemorrhage. *Clin Radiol* 1986; 37:193–4.
